# Hyaluronic Acid Improves Stability in Ovalbumin-Tea Polyphenol Pickering Particle-Stabilized Gel-like HIPEs via Interfacial Reinforcement

**DOI:** 10.3390/gels12050425

**Published:** 2026-05-13

**Authors:** Jingchun Ma, Shenghui Bi, Xue Yang, E Zhao, Ying Zhou, Chun Ye, Yuanyuan Liu, Qiujin Zhu

**Affiliations:** Guizhou Key Laboratory of New Quality Processing and Storage of Ecological Specialty Food, School of Liquor and Food Engineering, Guizhou University, Guiyang 550025, China; ma_jc8440@163.com (J.M.); 1215331352bzh@163.com (S.B.); 17785803948@163.com (X.Y.); 15283904696@163.com (E.Z.); zhying_0525@163.com (Y.Z.)

**Keywords:** hyaluronic acid, ovalbumin, tea polyphenols, high-internal-phase gel-like emulsions, stability, in vitro digestion

## Abstract

Protein-stabilized high-internal-phase Pickering gel-like emulsions (HIPGEs) have gained broad attention in the food industry and functional food sectors. Polyphenol–protein synergy is a common strategy to improve gel-like emulsion stability, yet issues such as insufficient interfacial viscosity persist, leading to poor long-term stability. Therefore, this study employed ovalbumin (OVA)-tea polyphenol (TP) as a composite model and introduced strongly negatively charged hyaluronic acid (HA) to construct a ternary Pickering gel-like emulsion with enhanced interfacial viscosity. We investigated the microstructure, physicochemical properties, stability mechanism, and simulated digestion behavior of the system. Results show that HA interacts with proteins and polyphenols via hydrogen bonding, strengthening the hydrogen-bond network and markedly improving gel-like emulsion stability. Moreover, HA stabilizes the oil–water interface by enhancing the viscoelasticity of the system. At 0.8% HA, centrifugal stability reached 99.52%, rheological properties were optimal, and droplets were more uniform and tightly packed. In vitro digestion revealed that 0.8% HA increased the final retention of lutein to 35.16% and reduced free fatty acid release to 0.31 μmol, demonstrating excellent protective and controlled-release potential. This study confirms that HA can significantly improve the stability and digestively controlled release of OVA-TP Pickering gel-like emulsions, providing theoretical support for polysaccharides in enhancing protein–polyphenol composite Pickering systems.

## 1. Introduction

Pickering gel-like emulsions represent a multifunctional oil–water biphasic system that exhibits superior resistance to droplet coalescence stabilized by solid particles rather than synthetic surfactants, they are generally regarded as having lower biotoxicity than conventional surfactant-stabilized emulsions [1] and thus hold considerable promise for applications in food processing and nutrient delivery [2]. When the internal phase volume fraction exceeds 74%, the emulsion is classified as a high-internal-phase Pickering gel-like emulsion (HIPGE). Characterized by densely packed droplets and a gel-like structure, HIPGEs display markedly enhanced viscoelasticity and a substantially increased interfacial area compared to conventional emulsions. These attributes confer superior stability and effectively prevent sedimentation [3,4]. Traditional high-internal-phase emulsions rely on substantial quantities of low-molecular-weight surfactants to maintain stability by suppressing droplet flocculation and coalescence. However, current market and consumer preferences increasingly favor natural, sustainable, and palatable ingredients, thereby challenging the continued extensive use of synthetic surfactants in food and biomedical applications [5].

Ovalbumin (OVA) is a water-soluble globular protein composed of 385 amino acid residues. In its native state, hydrophobic amino acids are predominantly located within the internal structure, while hydrophilic amino acids are exposed on the surface. This structural characteristic makes OVA an excellent natural protein emulsifier [6,7]. Studies have indicated that native OVA exhibits the ability to stabilize HIPE in neutral solutions [8]. Natural OVA combines emulsion stability with high palatability, offering a superior alternative to conventional approaches that rely on substantial quantities of surfactants to maintain high-internal-phase emulsion stability. Furthermore, the incorporation of active ingredients such as polyphenols into composite formulations can synergistically enhance its interfacial properties. Polyphenolic compounds reinforce the oil–water interface film of OVA emulsions, enhancing stability [9]. As potent natural antioxidants, tea polyphenols (TPs) inhibit protein and lipid oxidation, preventing undesirable flavor and color changes during food storage, thereby improving quality and safety [10]. Ovalbumin-catechin conjugates exhibit high storage stability, resistance to lipid oxidation, and strong interfacial accumulation capacity [11]. Covalent complexes of lactoferrin with epigallocatechin-3-gallate (EGCG) form denser, more uniform microstructures with smaller droplet sizes and higher stability, effectively inhibiting fish oil oxidation [12].

However, protein–polyphenol complexes exhibit environmental sensitivity, with polyphenols potentially losing their antioxidant and anti-inflammatory functional activities during processing or storage due to oxidation, degradation, or excessive binding to proteins, thereby compromising the long-term stability of emulsions. Research indicates that ternary protein–polyphenol–polysaccharide particles not only enhance the physicochemical stability of HIPGEs through multi-component cross-linking but also confer multifunctionality [13]. Hyaluronic acid (HA) is a linear macromolecular mucopolysaccharide composed of D-glucuronic acid and N-acetyl-D-glucosamine alternately linked via β-1,3 and β-1,4 glycosidic bonds. This biopolymer is non-toxic, possesses excellent biocompatibility and biodegradability, and exhibits remarkable antioxidant activity and other biological functions [14]. As an endogenous metabolic component in the human body, HA is approved for use as a dietary supplement and can be administered orally [15]. Complexes formed between HA and saffron by-product proteins can stabilize HIPEs [16]. Similarly, HA–zein adsorbed particles can stabilize HIPEs with oil phase volume fractions up to 80%, enabling higher loading of active components [17]. However, the optimal functional concentration of HA within ternary complex systems and its corresponding structural evolution patterns remain unclear.

Although ternary protein–polyphenol–polysaccharide systems have been reported to stabilize high-internal-phase emulsions, two critical gaps remain. First, the optimal functional concentration of hyaluronic acid (HA) in such complexes and the corresponding structural evolution patterns have not been systematically elucidated. Second, most previous studies have focused solely on emulsion stability, with a lack of a validated demonstration of controlled nutrient delivery under gastrointestinal conditions. To address these gaps, this study is based on HA’s polyhydroxy structure, strong negative charge, and polymer chain characteristics. It is hypothesized that upon introduction into the OVA-TP composite system, HA can form a dense three-dimensional network structure with OVA-TP through intermolecular interactions such as hydrogen bonding. By leveraging its electronegativity and interfacial viscoelasticity, HA is expected to synergistically enhance the interfacial stability and overall structural strength of HIPGEs. This interaction is anticipated to yield multiple effects: at the physical level, significantly enhancing the gel-like emulsion’s long-term storage stability and rheological properties; at the functional level, effectively encapsulating and protecting active ingredients like lutein to achieve controlled sustained release in the gastrointestinal tract. This study aims to address the limitations of traditional protein–polyphenol composite emulsification systems with respect to long-term stability and controllable nutrient delivery. To validate this hypothesis, multi-scale characterization techniques were employed to analyze molecular interactions and microstructural evolution. Rheological measurements and low-field NMR were combined to quantitatively characterize macroscopic mechanical properties and water distribution patterns. Furthermore, an in vitro digestion model system was used to evaluate the gastrointestinal sustained release and protective efficacy of HIPGEs for lutein.

## 2. Results and Discussion

### 2.1. Characterization of OVA-TP-HA Complex

#### 2.1.1. Fourier Transform Infrared Spectroscopy (FTIR) Analysis of the OVA-TP-HA Complex

**Figure 1:**
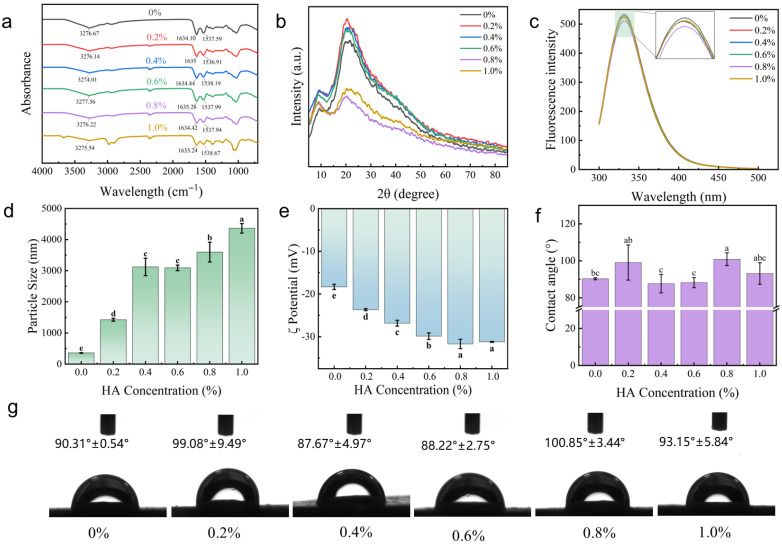
(**a**) FTIR spectrum of the complex, (**b**) XRD of the complex, (**c**) fluorescence spectrum of the complex, (**d**) particle size of the complex, (**e**) zeta potential of the complex, (**f**) contact angle of the complex, (**g**) images of the contact angle of the complex. Different lowercase letters indicate significant differences (*p* < 0.05).

FTIR was used to identify changes in functional groups and analyze interactions among OVA, TP, and HA [18]. All complexes exhibited the characteristic amide A band (3600–3000 cm^−1^), which was attributed to O–H and N–H stretching vibrations arising from intermolecular hydrogen bonds [19]. The addition of HA increased the infrared absorption intensity across all groups (Figure 1a), likely due to its strong hydrogen-bonding capacity and spatial network structure, which promoted non-covalent interactions with OVA and TP, thereby strengthening the intermolecular force network and forming a more stable, compact structure. The enhanced amide A band intensity further confirmed the enrichment of hydrogen bonds. Although the amide A band position shifted slightly (3274–3278 cm^−1^), it remained largely stable, indicating that HA reinforced and reorganized hydrogen bonding with minimal net energy change.

**Figure 2:**
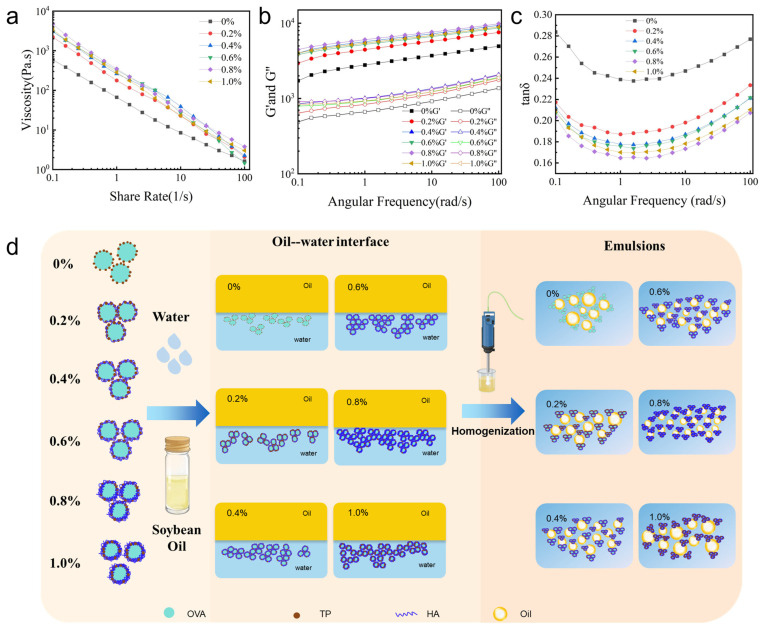
(**a**) Apparent viscosity of HIPGEs, (**b**) frequency scanning of HIPGEs, (**c**) tan δ of HIPGEs, (**d**) mechanism diagram of emulsion formation.

The 1700–1500 cm^−1^ region contains the amide I (1700–1600 cm^−1^, C=O stretching) and amide II (1600–1500 cm^−1^, coupled C–N stretching and N–H bending) bands [20]. In this system, HA reinforces the hydrogen-bond network via steric effects, restricting peptide backbone vibrations and causing a slight blue shift in the amide I band. At 0.2% HA, a slight red shift in the amide II band was observed, likely because strong hydrogen-bond acceptors on HA chains form hydrogen bonds with N–H groups on the OVA backbone, weakening the N–H bond and lowering its vibrational frequency. As HA concentration was increased further (0.4–1.0%), extensive HA chains were found to compete with proteins for binding sites and interact with each other, leading to a blue shift. The OVA-TP interaction relies mainly on weak hydrophobic forces or hydrogen bonds. As a key cross-linking component, HA promotes the formation of a broader and stronger hydrogen-bond network that encapsulates and immobilizes protein chains, thereby enhancing structural stability and suppressing molecular vibrations. These findings indicate that HA increases the cross-linking density and structural rigidity of the complex. Consistent with these FTIR results, the enhancement of the hydrogen-bond network upon HA addition directly accounts for the significant increase in storage modulus (G′) observed in rheological measurements (Figure 2b), indicating that molecular-scale interactions underpin the macroscopic viscoelastic gel network.

#### 2.1.2. X-Ray Diffraction Analysis (XRD) of the OVA-TP-HA Complex

The XRD patterns of the OVA-TP-HA complexes are shown in Figure 1b. All samples exhibited typical amorphous diffuse peaks at 2θ ≈ 20–25°, which are indicative of short-range order and long-range disorder in polymer chains [21]. Compared with the control (0% HA), the 0.2% HA sample showed a slight increase in peak intensity, likely due to localized ordered packing of OVA and TP molecules induced by functional groups on HA chains, which enhanced electron density fluctuations and X-ray scattering. However, as HA concentration was increased from 0.4% to 0.8%, peak intensity progressively decreased, reaching a minimum at 0.8%, indicating greater system disorder and disruption of short-range ordered regions. This may be attributed to steric hindrance arising from HA long-chain structures, which impede ordered packing, as well as increased viscosity, which restricts molecular motion, thereby collectively inhibiting crystallization [22].

#### 2.1.3. Fluorescence Spectral Analysis of the OVA-TP-HA Complex

Fluorescence spectroscopy, which is based on intrinsic aromatic amino acid fluorescence, enables non-invasive characterization of amino acid composition in composite materials under specific excitation wavelengths [23]. Each composite exhibited a maximum emission wavelength at 332 nm (Figure 1c), which is characteristic of OVA’s intrinsic fluorescence, which primarily originates from tryptophan residues. In this system, the fluorescence intensity of the OVA-TP-HA ternary composite gradually decreased with increasing HA concentration, likely due to binding near tryptophan residues that altered their microenvironment and hindered effective excitation or photon emission [24].

At 0.8% HA, fluorescence quenching was maximized, indicating that tryptophan residues were fully encapsulated within the complex network, with a concomitant increase in hydrophobicity and isolation from the aqueous environment, thereby reflecting the strongest interaction and most compact ternary structure at this concentration. At 1.0% HA, fluorescence intensity increased, likely due to steric hindrance and electrostatic repulsion among excess HA chains, which partially disrupted the compact network and re-exposed some buried residues to a more polar microenvironment, slightly reducing the quenching effect. These results confirm that OVA, TP, and HA form a stable ternary complex rather than a non-interacting physical mixture.

#### 2.1.4. Particle Size and Potential Analysis of the OVA-TP-HA Complex

OVA-TP without HA exhibited an average particle size of 359.43 nm (Figure 1d), which significantly increased to 4361.67 nm with increasing HA concentration (*p* < 0.05). OVA and TP initially form smaller particles via hydrophobic interactions and hydrogen bonding, while HA subsequently binds multiple OVA-TP particles through hydrogen bonds, forming larger aggregates. Larger solid particles provide superior kinetic stability at the oil–water interface due to higher desorption energy [25].

Zeta potential is a key indicator of complex formation, reflecting surface charge changes; higher absolute values enhance colloidal stability via stronger repulsive forces [26]. Under pH conditions above OVA’s isoelectric point, OVA-TP complexes consistently show negative zeta potentials (Figure 1e). As HA concentration increased (0–0.8%), the potential decreased from −18.3 mV to −31.7 mV due to increased negative charge density arising from HA’s charged groups. Moreover, research indicates that electrostatic complexes, formed by proteins and anionic polysaccharides bearing net negative charges, can adsorb to the oil–water interface. The resulting protective layer substantially enhances gel-like emulsion stability [27]. At 0.8% HA, the maximum zeta potential magnitude was achieved, indicating optimal electrostatic repulsion and colloidal stability. At 1.0% HA, the potential slightly decreased to −31.2 mV, though the change was not significant (*p* > 0.05), which is consistent with Wang et al. [28], suggesting that excess HA may moderately alter the interfacial dielectric environment or ion distribution without disrupting charge stability.

#### 2.1.5. Triphasic Contact Angle Analysis of the OVA-TP-HA Complex

The three-phase contact angle (θ) reflects particle wetting at oil–water interfaces: larger angles indicate higher hydrophobicity, while smaller angles indicate higher hydrophilicity. At θ = 90°, particles exhibit amphiphilicity, enabling effective adsorption and Pickering gel-like emulsion stabilization [29]. At 0.2% HA, the contact angle increased from 90.31° to 99.08° (Figure 1f,g), suggesting enhanced hydrophobicity due to HA–protein interactions exposing hydrophobic groups. At 0.4–0.6% HA, the angle decreased to 88.22°, as incorporated hydrophilic HA formed hydrogen-bond networks that exposed hydrophilic groups. At 0.8% HA, the contact angle increased again, likely because increased viscosity restricted molecular rearrangement, preventing the system from reaching a thermodynamically stable hydrophilic configuration during film formation. Research indicates that when composites form on solid surfaces, materials spontaneously minimize surface free energy to achieve thermodynamically stable states [30]. In aqueous environments, minimizing surface free energy requires maximizing surface hydrophobicity [31], with highly hydrophobic particles exhibiting superior interfacial activity [32].

#### 2.1.6. Transmission Electron Microscopy (TEM) Analysis of the OVA-TP-HA Complex

**Figure 3:**
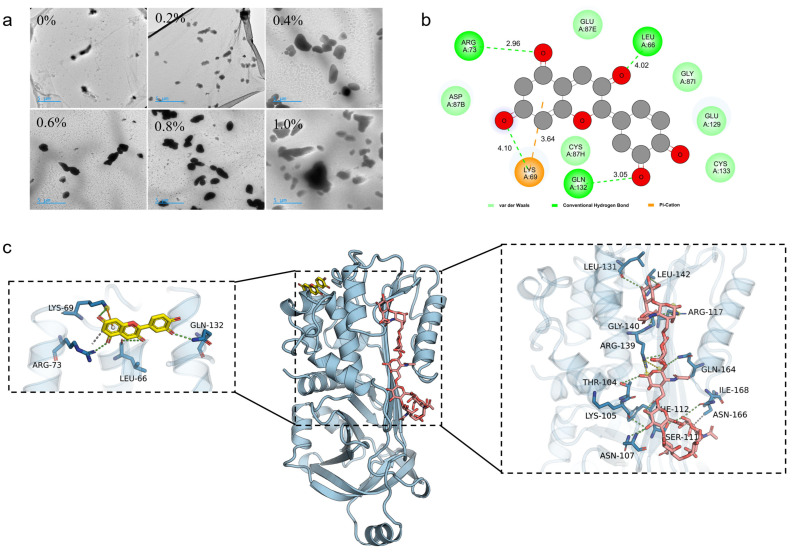
(**a**) TEM image of the complex (scale: 5 μm), (**b**) schematic diagram of two-dimensional molecular docking for OVA, Cat, and HA, (**c**) schematic diagram of three-dimensional molecular docking for OVA, Cat, and HA.

TEM images (Figure 3a) show that particle size increases with HA addition, consistent with particle size analysis. This is attributed to HA long-chain structures bridging multiple OVA-TP particles, thereby promoting aggregation or three-dimensional network formation. As a polymeric macromolecule, HA exhibits a strong hydrodynamic volume and chain entanglement [33]. With increasing HA concentration, intermolecular entanglement and cross-linking are intensified, embedding protein particles within a denser HA network and synergistically increasing overall complex size [34].

### 2.2. Molecular Docking of OVA, Catechin (Cat), and HA 

**Figure 4:**
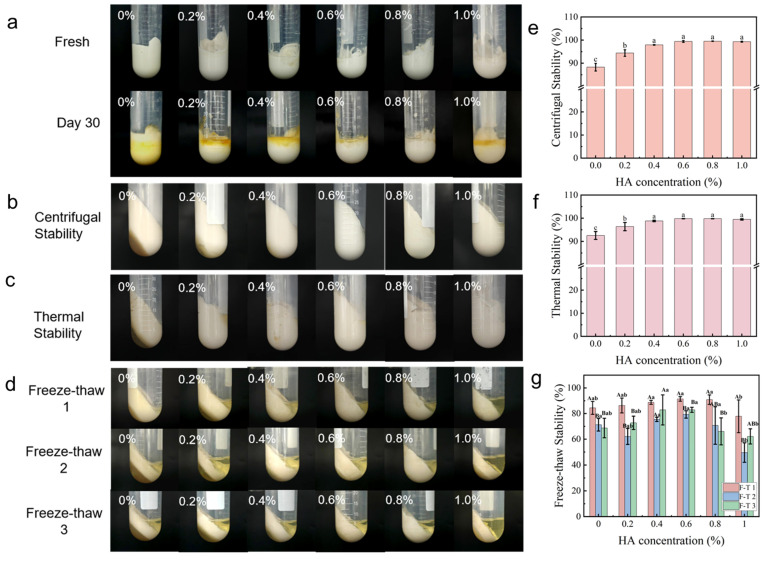
(**a**) Appearance of freshly prepared HIPGEs and HIPGEs after 30 days of storage, (**b**) appearance of HIPGEs after centrifugation, (**c**) appearance of HIPGEs after heating centrifuge, (**d**) appearance of HIPGEs after three freeze–thaw cycles, (**e**) centrifugal stability index of HIPGEs, (**f**) thermal stability index of HIPGEs, (**g**) freeze–thaw stability index of HIPGEs. Capital letters indicate differences in freeze–thaw stability between different cycle numbers within the same experimental group. Lowercase letters indicate differences in stability between different experimental groups at the same freeze–thaw cycle number. Different combinations of uppercase and lowercase letters indicate significant differences (*p* < 0.05).

Molecular docking simulations were employed to investigate the interactions among OVA, catechin (Cat, the primary component of TP), and HA (Figure 3b,c). Cat exhibited a highly complementary local binding profile. Its stable binding free energy (−7.189 kcal/mol) primarily originated from a directional hydrogen-bond network with key residues within the binding pocket (e.g., GLN132, ARG73, LYS69), supplemented by an n–cation interaction mediated by LYS69 and hydrophobic contacts involving ASP0. This configuration promotes tight encapsulation and conformational locking of the ligand within the hydrophobic core of the protein. In contrast, HA displayed a typical multivalent, global binding mode. Its stability relies on an extensive hydrogen-bond interaction network distributed across the protein surface (involving more than ten residues such as PHE112, LYS105, ARG117, and ARG139), which is notably reinforced by an intermolecular salt bridge formed between ARG117 and ARG139. This multipoint anchoring mechanism enables HA to effectively cover and bind to the protein surface through strong polar and electrostatic interactions. These docking results are consistent with experimental observations: HA’s multiple hydrogen bonds with OVA account for the enhanced amide A band and blue shift of the amide I band in FTIR, while local electrostatic interactions account for changes in the tryptophan microenvironment observed in fluorescence spectroscopy. Overall, Cat binding reflects high shape and chemical complementarity with the active pocket, whereas HA binding resembles surface adsorption and stabilization via multivalent effects, providing biophysical insights for functional studies. Furthermore, the binding energies and stable interaction sites predicted by molecular docking correlate well with the observed long-term storage stability (Figure 4a), as strong interfacial binding prevents droplet rearrangement and coalescence over time.

### 2.3. Physical Stability of HIPGEs

#### 2.3.1. Storage Stability

During gel-like emulsion storage, stability is often compromised by oil phase separation, with oil leakage serving as a key indicator of storage stability. All gel-like emulsions were initially uniform (Figure 4a). After 30 days of static storage, varying degrees of oil phase separation were observed. For the group without HA (0% group), the emulsion turned from white to yellow accompanied by oil phase separation, which may be attributed to the inherent instability of the matrix components in the absence of HA protection. Over the 30-day storage period, oxygen permeation led to oxidation of protein side chains and phenolic compounds, producing yellowish-brown products [35]. With increasing HA concentration (0.2–0.6%), oil phase separation gradually decreased and emulsion stability increased. The addition of HA can enhance the rigidity of the OVA-TP interfacial film, reduce interfacial tension, and increase the viscosity of HIPGEs (Figure 2a), thereby inhibiting Ostwald ripening and flocculation, and improving the physical stability of the emulsion [36]. At 0.8% HA, minimal separation was observed, attributable to balanced steric and electrostatic stabilization from the optimal OVA-TP-HA network. The tightly packed OVA–TP–HA particles at the oil–water interface form a robust steric barrier; this dense particle layer effectively prevents droplet coalescence by increasing the energy barrier to film thinning, which constitutes a hallmark of the Pickering stabilization mechanism (Figure 2d). At 1.0% HA, stability declined as high viscosity hindered homogenization, resulting in larger droplets prone to destabilization.

#### 2.3.2. Centrifugal Stability

Centrifugal stability predicts a gel-like emulsion’s resistance to gravitational separation during storage [37]. After centrifugation (Figure 4b), HA-free HIPGEs (0%) showed aqueous phase separation due to insufficient OVA-TP network strength. With increasing HA (0.2–0.6%), separation decreased and centrifugal stability improved (Figure 4e), which was attributed to enhanced network viscosity (Figure 2a) that immobilizes water and traps oil droplets, suppressing creaming and coalescence. At 0.8% HA, centrifugal stability peaked at 99.52%, indicating an optimal network resistant to centrifugal stress. At this point, HA is likely sufficient to fully cover the OVA molecules, forming a uniform composite network with adequate steric hindrance and electrostatic repulsion without disrupting the overall balance. This optimized interfacial layer can most effectively prevent droplet coalescence and phase separation, thereby endowing the emulsion with optimal stability [38].

#### 2.3.3. Thermal Stability

Good thermal stability is crucial for the food application of gel-like emulsions [39]. The thermal stability of HIPGEs showed the same trend as their centrifugal stability (Figure 4c,f). Increasing HA from 0% to 0.6% raised thermal stability from 92.56% to 98.80%, owing to HA’s water-holding capacity and high viscosity in suppressing heat-driven droplet movement and phase separation. At 0.8% HA, thermal stability peaked at 99.77%, indicating optimal packing of the OVA-TP-HA ternary system, which maximizes steric hindrance and viscoelastic support. Heated HIPGEs released less aqueous phase and oil phase upon centrifugation than unheated samples (Figure 4b,c), suggesting a thermal responsive network where heating enhances stability, likely due to OVA denaturation and aggregation that further strengthening the three-dimensional structure and restricting phase migration [40].

#### 2.3.4. Freeze–Thaw Stability

Freeze–thaw stability is a key performance metric for frozen food gel-like emulsions. Phase separation occurred in all HIPGE samples after freeze–thaw cycling (Figure 4d), indicating sensitivity to this treatment. Stability after the first cycle was significantly higher than after subsequent cycles (*p* < 0.05) (Figure 4g), as repeated cycles caused progressive structural damage. At low HA concentrations (0–0.4%), freeze–thaw stability was poor due to insufficient steric hindrance and viscoelastic buffering against ice crystal formation. At an HA concentration of 0.6%, the freeze–thaw stability was enhanced. This may be attributed to the strong hydrophilicity and high water-holding capacity of HA, which enable it to form a non-ice-phase-enriched layer in the aqueous phase during the freezing process. This layer can buffer the squeezing effect of ice crystals on the emulsion droplets, thereby improving the freeze–thaw stability to a certain extent [38]. At 0.8% HA, HIPGEs maintained relatively good stability after the first cycle (90.83%), but the network may have been overly rigid (highest G′ value, Figure 2b), making it prone to micro-cracks or brittle failure under repeated freeze–thaw stress, leading to reduced stability after multiple cycles [41]. Nevertheless, the 0.8% HA group exhibited the best overall performance in terms of storage stability, centrifugal stability, and other indicators.

### 2.4. Microstructure of HIPGEs

**Figure 5:**
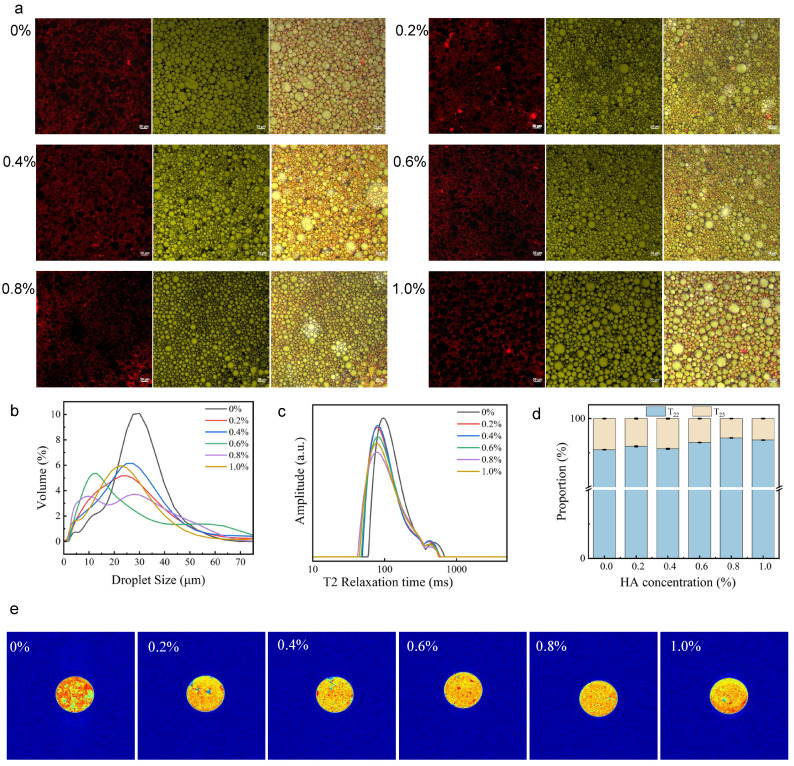
(**a**) CLSM images of HIPGEs (scale: 20 μm), (**b**) particle size distribution of HIPGEs, (**c**) T_2_ relaxation curve of HIPGEs, (**d**) T_22_ and T_23_ Relaxation Percentages of HIPGEs, (**e**) nuclear magnetic resonance imaging of HIPGEs.

CLSM images (Figure 5a) show that HIPGEs are of the O/W type, with oil droplets (green) surrounded by a protein network (red) in the continuous phase [42]. As HA concentration was increased, oil droplets became smaller, likely because HA enhances emulsifier adsorption at the oil–water interface and inhibits coalescence via steric hindrance [28]. At 0.8% HA, droplets were smallest and most uniform, which improved stability through increased specific surface area and Brownian motion [43], while also contributing to a compact internal structure that enhances rheological strength [44]. At 1.0% HA, droplet size increases, as high viscosity hinders shear transfer and reduces emulsification efficiency.

### 2.5. Particle Size of HIPGEs

Droplet size distribution reflects gel-like emulsion stability. Without HA, the distribution was unimodal with a high-intensity peak centered at ~30 μm (Figure 5b), likely due to insufficient stability of the OVA-TP-stabilized gel-like emulsion, leading to aggregation into large, uniform droplets. As HA concentration was increased (0.2–0.6%), the peak shifted to smaller sizes, with reduced height and increased width, indicating that the addition of HA strengthens the interfacial film, promoting droplet breakup during homogenization to form smaller droplets, thereby reducing the average droplet size of the gel-like emulsion [38]. At 0.8% HA, a bimodal distribution emerged: a main peak at ~9 μm from finely sized, uniform droplets stabilized by the OVA-TP-HA interfacial film, which forms a dense three-dimensional coating on droplet surfaces, and a secondary peak at ~27 μm, likely representing droplet clusters or flocs formed via the HA-mediated three-dimensional network. At 1.0% HA, the peak shifted to larger sizes, attributable to excessive aqueous phase viscosity that reduced homogenization efficiency and an overly cross-linked network that resulted in larger, more compact droplets.

### 2.6. Moisture Distribution in HIPGEs

LF-NMR is employed to analyze the oil–water distribution in gel-like emulsions, which reflects the internal structure and protein interactions. Hydrogen proton mobility is governed by the density of the aqueous network, with tighter structures imposing greater confinement [45]. The T_2_ relaxation time is sensitive to proton migration and serves as a key indicator of molecular motion; shorter T_2_ times indicate stronger proton confinement [46]. The absence of a T_21_ peak in the relaxation curve (Figure 5c) may be attributed to most hydrogen protons being tightly confined within the OVA-TP-HA three-dimensional network, rendering the T_21_ signal too weak to appear as a distinct peak. As HA concentration was increased (0–0.8%), both T_23_ and T_22_ peaks shifted to shorter relaxation times, indicating a more compact network with stronger proton restriction. Additionally, the proportion of the T_23_ component decreased while that of T_22_ increased (Figure 5d), suggesting that HA incorporation enhanced interfacial strength and aqueous phase cross-linking, converting some free protons to an immobilized state, which is consistent with Sun et al. [16].

Without HA (0%), LF-NMR imaging (Figure 5e) showed large aggregated red spots, indicating significant free-state regions and uneven hydrogen proton distribution. This was attributable to the absence of steric stabilization by HA, leading to low continuous phase viscoelasticity and ineffective proton immobilization. As HA concentration was increased (0.2–0.6%), proton distribution gradually became more uniform. At 0.8% HA, imaging revealed a uniform proton distribution, attributed to a uniform HA-OVA-TP network encapsulating and immobilizing protons. At 1.0% HA, localized red spot aggregation reappeared, likely due to HA self-association and excessive entanglement, disrupting the uniform network and causing proton expulsion.

### 2.7. Rheological Properties of HIPGEs

As shown in Figure 2a, all HIPGEs exhibit pseudoplastic shear-thinning behavior, in which apparent viscosity decreases with increasing shear rate [47]. As HA concentration was increased from 0% to 0.8%, viscosity progressively rose, reaching a maximum of 4727.14 Pa·s at 0.8% HA due to HA chain entanglement and the formation of a three-dimensional network [25]. This network not only markedly increases the bulk viscosity of the continuous phase but also effectively inhibits Brownian motion, coalescence, and Ostwald ripening of oil droplets, thereby significantly improving the macroscopic physical stability of the emulsion [28]. Moreover, the dense negative charges on the HA molecular chains enhance the electrostatic repulsion within the entire system, further preventing droplet approach [48]. However, viscosity decreased at 1.0% HA, likely due to reduced homogenization efficiency under high-viscosity conditions, leading to larger residual oil droplets and compromised gel-like emulsion stability and viscoelasticity.

The storage modulus (G′) characterizes the elastic (solid-like) behavior of a material, quantifying the energy stored during elastic deformation. By contrast, the loss modulus (G″) characterizes the viscous (liquid-like) behavior, representing the energy dissipated as heat during viscous flow [49]. Across the frequency range, both G′ and G′′ values for HIPGEs increase with rising angular frequency (Figure 2b). Within the 0.1–100 rad/s frequency range, HIPGEs exhibit a significantly higher G′ than G′′, indicating that elasticity dominates within the viscoelastic and robust network structure of HIPGEs. This enables effective resistance to deformation induced by external stresses, thereby suppressing destabilizing processes such as oil droplet migration, flocculation, and coalescence [50]. Both the storage modulus (G′) and loss modulus (G′′) of the HIPGEs rose markedly with HA addition, peaking at a concentration of 0.8% HA (G′ = 9843.08 Pa, G′′ = 2041.47 Pa), corresponding to the smallest and most uniform droplets observed by CLSM (Figure 5a). This indicates that HA-OVA-TP forms a dense interfacial film through multivalent hydrogen bonding (Figure 3b,c), and that HIPGEs containing 0.8% HA exhibit excellent shape retention and energy dissipation capabilities, maintaining structural integrity under substantial stress with a more stable network and enhanced resistance to deformation forces.

**Figure 6:**
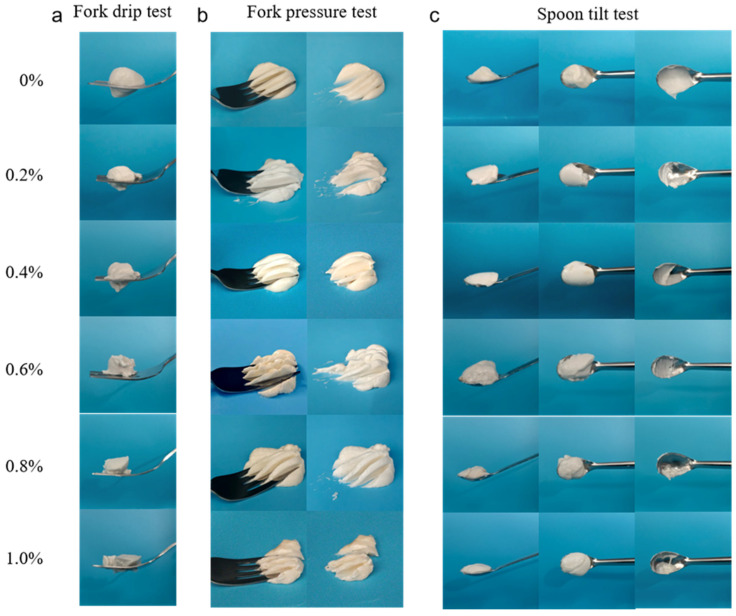
(**a**) Fork drip test, (**b**) fork pressure test, (**c**) spoon tilt test for HIPGEs.

The rate of increase in G′ far exceeded that of G′′, resulting in a continuous decline in the tan δ ratio. The tan δ values for all samples ranged between 0.1 and 0.3 (Figure 2c), indicating that the interfacial layer was predominantly elastic. With increasing HA content, tan δ values markedly decreased, reaching a minimum of 0.16 at 0.8% HA addition—corresponding to the highest viscosity (Figure 2a). This likely arises from HA cross-linking with OVA-TP via hydrogen bonds, forming a dense three-dimensional network that promotes elastic energy storage rather than dissipation within HIPGEs. A lower tan δ indicates reduced energy dissipation within the system, enhanced structural recovery capacity, and a greater tendency toward stable dispersion [36], which is crucial for long-term storage stability. At 0.8% HA concentration, tan δ reached its minimum, signifying that the OVA-TP-HA system can form a rapidly reconfigurable physical cross-linked network. This network is capable of swiftly restoring its original structure even after shear disruption, thereby enhancing the gel-like emulsion’s physical stability. Furthermore, the fact that the sample deforms without breaking in the IDDSI fork pressure test demonstrates that HIPGEs can rapidly recover their original structure after shear disruption (Figure 6b).

HA binds to the OVA surface through multiple hydrogen bonds and salt bridges, while catechin embeds into the hydrophobic pocket of OVA via a directional hydrogen-bond network, forming a stable OVA-TP-HA ternary complex (Figure 3b,c). The enhancement of the amide A band and the blue shift of the amide I band in FTIR spectra confirmed that HA strengthened the overall hydrogen-bond network (Figure 1a), while the gradual quenching of tryptophan residues in fluorescence spectra (maximum at 0.8% HA) indicated an enhanced hydrophobic microenvironment and tighter embedding of the peptide chain upon formation of the ternary complex (Figure 1b). This cross-linked structure at the molecular level enables the HA-OVA-TP complex to exhibit higher adsorption efficiency and a denser arrangement at the oil–water interface (Figure 2d), thereby effectively reducing the droplet size of the emulsion [51]. When the volume fraction of the dispersed phase exceeds 74%, the particle structure exhibits a positive correlation with the rheological properties of the gel-like emulsion [52]. Typically, smaller particles can more effectively stabilize the gel-like emulsion interface, forming a denser interfacial film that confers higher viscosity and storage modulus on the gel-like emulsion while enhancing emulsion stability [53]. Simultaneously, HA exhibits strong hydrophilicity and steric hindrance effects. Its extended chains within the aqueous phase generate significant spatial repulsion, preventing coalescence of adjacent oil droplets induced by van der Waals forces. Furthermore, HA indirectly promotes stable dispersion of fine oil droplets by increasing the viscosity of the continuous phase. The high-viscosity environment suppresses Brownian motion and reduces the collision frequency of oil droplets, preventing the dissolution and redeposition of small droplets onto larger droplet surfaces [54]. However, this effect exhibits concentration dependence: as HA concentration increases, droplet size progressively decreases. At 0.8% HA concentration, droplets reach their smallest size; beyond this, increasing HA concentration to 1.0% causes droplet size to increase (Figure 2a). Thus, HA forms stable complexes with OVA-TP through non-covalent interactions, simultaneously enhancing interfacial adsorption behavior and regulating bulk rheological properties, thereby achieving effective control of gel-like emulsion droplet size.

### 2.8. IDDSI

IDDSI provides a global standard for assessing food swallowing difficulty. In the fork drip test (Figure 6a), HIPGEs with HA concentrations ≥0.6% accumulate on the fork and fail to drip or flow through the fork’s prongs or tip without trailing, which is characteristic of level 4 paste-like/extremely thick foods [55]. Similar to the pressure applied by a fingertip, the tongue exerts a force of approximately 17 kPa during swallowing [56]. As shown in Figure 6b, all HIPGEs crushed and deformed readily under minimal pressure, leaving no surface lumps and forming distinct impressions that did not revert to their original shape upon fork removal. These results indicate that the texture of all samples meets the criteria for being easily portioned under gentle pressure, making them appropriate for utensil-assisted eating and manageable for those with chewing difficulties associated with dysphagia [57]. The spoon tilt test (Figure 6c) reflects surface smoothness and cohesive strength related to integrity [58]. All HIPGEs possess sufficient cohesive strength to maintain their original shape when the spoon is tilted. When inverted and subjected to force, substantial detachment of HIPGEs occurred. Gel-like emulsions without HA formed a thin film on the spoon surface, with residue decreasing as HA content increased. At 0.8% HA, the gel-like emulsion exhibited significant textural advantages, leaving minimal residue and ensuring no sticky sensation on the tongue or throat.

### 2.9. In Vitro Digestion

#### 2.9.1. Appearance and Microstructure

**Figure 7:**
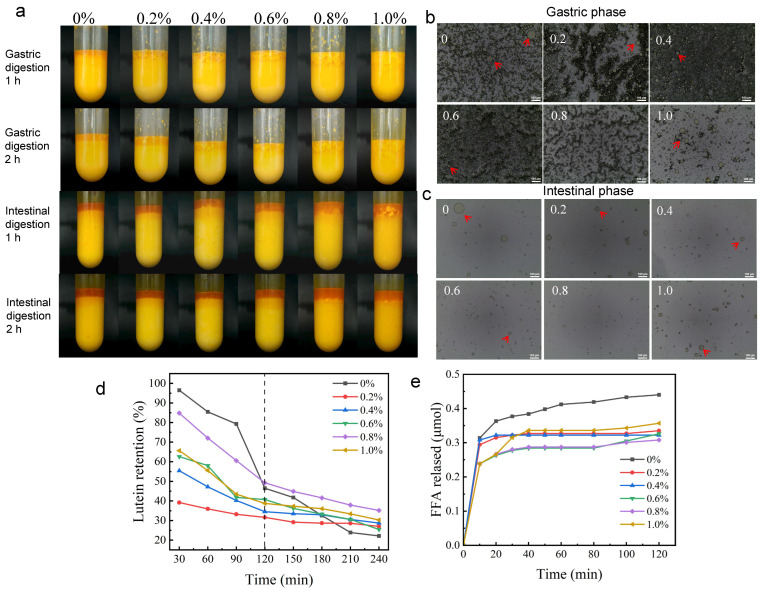
(**a**) Appearance of HIPGEs encapsulating lutein during digestion, (**b**) optical microscope image of HIPGEs encapsulating lutein after gastric digestion (scale: 100 μm, the arrows point to the large droplets), (**c**) optical microscope image of HIPGEs encapsulating lutein after intestinal digestion (scale: 100 μm, the arrows point to the large droplets), (**d**) lutein retention rate during simulated in vitro digestion, (**e**) free fatty acid release.

As digestion time was prolonged, gel-like emulsions gradually exhibited stratification and phase separation (Figure 7a), with higher HA concentrations delaying the onset of stratification. HA enhanced gastric phase stability by increasing aqueous phase viscosity and providing steric hindrance, inhibiting droplet coalescence and buoyancy. During gastric digestion, HIPGEs without HA addition (0% group) exhibited isolated larger droplets (Figure 7b). With increasing HA concentration, droplets were dispersed uniformly without noticeable large aggregates. This may be attributable to HA’s steric hindrance effect establishing physical barriers between droplets, coupled with HA’s ability to maintain its hydration capacity and network structure under acidic conditions, thereby sustaining system stability.

During intestinal digestion, the number of gel-like emulsion droplets markedly decreased (Figure 7c), indicating lipid breakdown by intestinal fluids [59]. Bile salts and lipase adsorb onto droplet surfaces, hydrolyzing triglycerides and causing structural disintegration. Without HA (0% group), isolated large droplets were observed, likely due to the fragile OVA-TP bilayer membrane rapidly undergoing penetration and complete hydrolysis, leading to oil release and coalescence. With increasing HA (0.2–0.8%), a more robust composite membrane was formed with OVA-TP, effectively delaying enzyme action, maintaining droplet integrity and uniform size, and preventing coalescence. At 0.8% HA, the smallest and most uniformly distributed droplets were observed, indicating the most controlled and gradual lipid release, thereby providing an ideal structural foundation for efficient active ingredient delivery.

#### 2.9.2. Lutein Retention Rate

During digestion, the retention rate of lutein exhibited a trend of rapid initial decrease followed by a gradual decline with increasing digestion time (Figure 7d). The initial rapid loss of lutein is attributed to surface-attached lutein, which is immediately exposed to digestive enzymes and oxidative factors. The subsequent slow phase corresponds to lutein protected within the oil phase, which requires disruption of the interfacial film and digestion of lipids before release. In the group without HA, the lutein retention rate decreased from 96.50% to 22.13%, revealing the inherent deficiency of the Pickering interfacial film formed solely by OVA-TP particles in the complex digestive environment; namely, the system lacks effective protection for lutein. HIPGEs with HA (0.2–0.6%) exhibited higher final retention rates than the HA-free group, which was attributed to the three-dimensional network formed by HA that increased mass transfer resistance, restricted droplet mobility, and reduced enzyme diffusion and activity. HIPGEs with 0.8% HA achieved the highest final lutein retention rate of 35.16%. This is likely because at this concentration (0.8%), HIPGEs exhibit the highest viscoelastic modulus (Figure 2b) and structural stability (Figure 4a), and the three-dimensional network reaches an optimal crosslinking state, providing not only the strongest bulk-phase mass transfer barrier but also regulating the rate of lipid digestion and lutein release in the intestine, avoiding massive release and degradation over a short time while retaining more active substances at the endpoint [60].

#### 2.9.3. Total Free Fatty Acid Release

Most lipid digestion occurs in the small intestine, where free fatty acid (FFA) release is measured [61]. During first 10 min of digestion, FFA was rapidly released, then gradually leveled off (Figure 7e). This is because the emulsified oil droplets are initially exposed to high concentrations of digestive enzymes, leading to rapid hydrolysis; as interfacial lipids are consumed or the interfacial structure is remodeled, the reaction rate gradually decreases. The control group without HA (0% HA) exhibited the highest cumulative FFA release, directly reflecting the fragility of its interfacial protection mechanism. As HA concentration increased, the amount of free fatty acids released decreased. This is attributed to HA forming a dense interfacial membrane with OVA-TP and a three-dimensional network in the aqueous phase, which increases viscosity (Figure 2a), restricts droplet movement, reduces bile salt and lipase adsorption, and delays digestion [62], while high viscosity further impedes enzyme penetration [63]. At an HA concentration of 0.8%, the FFA release reached the lowest level at 0.31 μmol, indicating that HIPGEs with 0.8% HA possess the highest viscoelastic modulus and greatest stability, thereby providing the strongest barrier against digestive enzymes and achieving an optimal effect in slowing down fatty acid release.

## 3. Conclusions

This study systematically constructed and thoroughly investigated a high-internal-phase gel-like emulsion stabilized by an OVA-TP-HA ternary complex and evaluated its potential as a functional delivery carrier for lutein. The results demonstrated that HA, through its excellent hydrogen-bonding capacity, forms non-covalent interactions with OVA and TP, collaboratively creating a tighter and more stable complex structure. The addition of 0.8% HA yielded the most optimal improvement in the stability of the OVA-TP-HA composite Pickering gel-like emulsion. It stabilizes the dispersion characteristics of the system by increasing negative charge to enhance repulsion between droplets. Furthermore, HA increased the viscoelasticity of the gel-like emulsion system, improved interfacial cross-linking strength, and promoted the stable existence of the Pickering gel-like emulsion. Meanwhile, HIPGEs with 0.8% HA were most effective in delaying lipid digestion, in better protecting lutein during intestinal digestion, in reducing free fatty acid release, and in demonstrating excellent protective performance and sustained-release potential. Theoretically, this study provides a paradigm for the targeted design of colloidal network properties through precise polysaccharide concentration regulation. Practically, it offers a clear formulation strategy and a solid scientific foundation for developing high-performance nutrient delivery products based on natural ingredients, demonstrating significant industrial potential.

## 4. Materials and Methods

### 4.1. Materials

OVA (75% purity, solubility 40 mg/mL, Catalog No. CAS9006-59-1) was procured from Shanghai Yuanye Company (Shanghai, China). TP (95% purity, food-grade) was purchased from Guangzhou Tianyuan Biological Company (Guangzhou, China). HA (molecular weight 800,000–1,500,000, Catalog No. CAS 9004-61-9) and lutein (purity 75%, 98%) were procured from Shanghai McLean Company (Shanghai, China). Soybean oil was purchased from a Walmart supermarket (Guiyang, China).

### 4.2. Preparation of the OVA-TP-HA Complex

The OVA powder sample was dissolved in distilled water to achieve a concentration of 3% (*w*/*v*). The dispersion was stirred continuously at 25 °C (500 rpm) until complete dissolution was achieved. The dissolved OVA dispersion was then placed at 4 °C overnight to ensure full hydration. Subsequently, the pH of the OVA dispersion to 7, then add 1% TP to prepare the OVA-TP complex solution. Accurately weigh 0, 0.2, 0.4, 0.6, 0.8, and 1.0 g of HA samples were weighed accurately and added to 100 mL of OVA-TP complex solution respectively. Stirring was continued for 2 h to obtain OVA-TP-HA solutions with HA concentrations of 0.2%, 0.4%, 0.6%, 0.8%, and 1.0% (*w*/*v*).

### 4.3. Characterization of the OVA-TP-HA Complex

Fourier-transform infrared spectroscopy (Frontier, PerkinElmer, Waltham, MA, USA) was employed to collect spectral data on all samples. Phase analysis of freeze-dried OVA-TP-HA composite samples was performed by (X-ray diffraction) XRD (Cu Ka radiation, 40 kV, 2θ range of 5–85°). Fluorescence spectroscopy (F-2700, Tokyo, Hitachi, Japan) was conducted with an excitation wavelength of 280 nm, recording spectra from 300 to 500 nm. The droplet size and zeta potential of the complex were determined using a nanoparticle size and potential analyzer (Delsa Nano C, Beckman Coulter, Brea, CA, USA), with all measurements conducted at 25 °C. The freeze-dried powders were pressed into tablets, and the three-phase contact angle was determined with a contact angle meter (DSA25, DKSH, Zurich, Switzerland). The microstructure of the composite particles was observed using a transmission electron microscope (JEOL Ltd., Tokyo, Japan) at an accelerating voltage of 160 kV.

### 4.4. OVA, Catechin (Cat), HA Molecular Docking

Molecular docking was employed to directly visualize the intermolecular interactions between HA and OVA, as well as catechin (the primary component of TP, Cat). The ovalbumin protein structure was obtained from the PDB database (PDB ID: 1OVA), with crystallization water, salt ions, and small-molecule ligands removed during preprocessing. The 3D structures of Cat and HA were visualized using Avogadro2 and minimized using the MMFF94 force field. Using ADFR suite 1.0, a docking box covering the entire protein was generated. Auto Dock Vina 1.2.7 was employed to perform the docking, with an exhaustiveness value set to 8 and other parameters maintained at default settings, after the receptor and ligand were converted to PDBQT format. The highest-scoring conformation was selected, and intermolecular interactions were visualized and analyzed using PyMOL 2.5.5.

### 4.5. Preparation of HIPGEs

The aqueous phase consisted of an OVA-TP-HA complex dispersion system, accounting for 25% (*v*/*v*) of the total volume, while soybean oil comprised the oil phase, making up the remaining 75% (*v*/*v*). The oil–water mixture was then homogenized continuously for 1 min at 12,000 rpm using a high-speed disperser (model XHF-DY, Xinzhi, Ningbo, China) to form the final HIPGEs.

### 4.6. Physical Stability Analysis of HIPGEs

HIPGEs were stored in sealed centrifuge tubes in a refrigerator at 4 °C for 30 days and photographed to record oil separation for storage stability assessment.

For centrifugal stability, freshly prepared HIPGEs were centrifuged at 5000 rpm for 15 min at 25 °C. Centrifugal stability was calculated as follows:

(1) $$Centrifugal\;Stability\left(\%\right)=\frac{W_{0}-W_{1}}{W_{0}}\times 100$$

where W_0_ denotes the total mass of HIPGEs prior to centrifugation, while W_1_ represents the combined mass of oil and water separated after centrifugation.

For thermal stability, HIPGEs were heated at 80 °C for 30 min, cooled to 25 °C, and stored overnight at room temperature (25 °C) before centrifugal stability testing.

For freeze–thaw stability, HIPGEs underwent three cycles of freezing (−20 °C for 22 h) and thawing (25 °C for 2 h), followed by centrifugal stability testing.

### 4.7. Confocal Laser Scanning Microscopy (CLSM) Analysis of HIPGEs

Imaging was performed using a confocal laser scanning microscope (LSM 900, Carl Zeiss, Jena, Germany) with a 20× objective lens. Observation was conducted using the same instrument.

### 4.8. Particle Size Analysis of HIPGEs

The droplet size was measured using a laser particle size analyzer (LS13320, Beckman Coulter, Brea, CA, USA). Samples were diluted in water and automatically dispersed until achieving optimal laser obscuration prior to measurement.

### 4.9. Low-Field Nuclear Magnetic Resonance (LF-NMR) Analysis of Moisture Distribution in HIPGEs

The transverse relaxation of HIPGEs was measured using an LF-NMR analyzer (NMI20-025V-I, Suzhou Niumag Co., Ltd., Suzhou, China). Each sample was placed in a sample vial and then inserted into a 25 mm NMR tube for testing. This analysis was employed to evaluate the moisture distribution and proton mobility within the HIPGEs.

### 4.10. Analysis of the Rheological Properties of HIPGEs

Rheological testing was conducted on a rheometer (TA HR10, Waters, New Castle, DE, USA). After loading, samples were equilibrated at 25 °C for 5 min. The linear viscoelastic region (LVR) was determined by dynamic strain sweep (0.1–100% strain, 1 Hz). Frequency sweeps (0.1–100 rad/s) were performed at 1% strain. Apparent viscosity was measured over a shear rate range of 0.1–100 s^−1^. Storage (G′) and loss (G″) moduli were obtained from oscillatory frequency sweeps (0.1–100 rad/s).

### 4.11. Experimental Analysis of the International Dysphagia Diet Standardization Initiative (IDDSI) by HIPGEs

The IDDSI provides an internationally recognized framework of terminology and assessment methods for texture-modified foods and thickened liquids. This framework categorizes these products into eight distinct levels (0–7). To assess safety levels, HIPGE samples were subjected to three tests: compression in the fork pressure test; scooping and lifting in the fork drip test; and scooping and then tilting in the spoon tilt test [64].

### 4.12. In Vitro Digestion Analysis of HIPGEs

Lutein (1.50 mg/mL) in soybean oil was added at 75% of the total volume to the complex solution and homogenized (12,000 rpm, 1 min). In vitro digestion was adapted from Minekus et al. [65].

Gastric stage: 20 mL of gel-like emulsion was mixed with 16 mL of pre-warmed SGF. The pH was adjusted to 2 using HCl (1 M) with CaCl_2_ (0.3 M, 1 μL). Then, 2 mL of pepsin solution (2000 U/mL) was added. Final volume was 40 mL (sample: SGF ratio 1:1). Samples were incubated at 37 °C and 200 rpm for 2 h.

Intestinal stage: 20 mL of gastric chyme mixture was collected. The pH was adjusted to 7.0 with NaOH (1 M) and distilled water (1.31 mL). Then, 11 mL of SIF, 2.5 mL of bile salt (10 mM), and 40 μL of CaCl_2_ (0.3 M) were added. Finally, 5 mL of pancreatin (4000 U/g) was introduced. Samples were incubated at 37 °C and 200 rpm for 2 h.

#### 4.12.1. Appearance and Optical Microscope Analysis of HIPGEs

Photographs of the gel-like emulsion during digestion were taken at hourly intervals to observe changes in appearance. The morphology of the gel-like emulsion at various stages of gastric and intestinal digestion was examined using an optical microscope.

#### 4.12.2. Analysis of Lutein Retention in HIPGEs

The organic extractant (n-hexane: ethanol = 3:2 *v*/*v*) was mixed with an equal volume (0.5 mL each) of HIPGEs and vortexed for 30 s to induce demulsification and extraction. After centrifugation at 4 °C and 12,000 rpm for 40 s, 50 μL of the supernatant was diluted with 950 μL of n-hexane, and the absorbance was measured at 450 nm using a microplate reader (Spectra MAX190, Molecular Devices, San Jose, CA, USA). Lutein content was determined using a calibration curve prepared with lutein standards in n-hexane.

(2) $$\begin{array}{c} \mathrm{Lutein}\mathrm{retention}\mathrm{rate}=\frac{\mathrm{Lutein}\mathrm{content}\mathrm{in}\mathrm{treated}\mathrm{gel}-\mathrm{like}\mathrm{emulsion}\mathrm{sample}}{\mathrm{Lutein}\mathrm{content}\mathrm{in}\mathrm{untreated}\mathrm{gel}-\mathrm{like}\mathrm{emulsion}\mathrm{sample}}\times 100\% \end{array}$$

#### 4.12.3. Analysis of Total Free Fatty Acid (FFA) Release During In Vitro Digestion

During simulated intestinal digestion, the pH of the digestive fluid was equilibrated using NaOH solution according to the pH-stat method [66]. Based on the formula below, the volume of NaOH consumed during digestion at 10, 20, 30, 40, 50, 60, 80, 100, and 120 min was used to calculate the release of free fatty acids (FFA) from the gel-like emulsion system.

(3) $$M_{FFA}=V_{NaOH}\times C_{NaOH}\times 1000$$

where M_FFA_ denotes the amount of FFA released, μmol; V_NaOH_ represents the volume of NaOH consumed during titration, L; and C_NaOH_ indicates the concentration of the NaOH solution, mol/L [67].

### 4.13. Statistical Analysis

All experiments were performed in triplicate. Data are expressed as mean ± standard deviation and analyzed using IBM SPSS Statistics 26. Intergroup differences were assessed by one-way ANOVA, with statistical significance set at *p* < 0.05.

## Data Availability

The original contributions presented in this study are included in the article Further inquiries can be directed to the corresponding authors.
